# Time-dependent risk of COVID-19 death with overwhelmed health-care capacity in Japan, 2020–2022

**DOI:** 10.1186/s12879-022-07929-8

**Published:** 2022-12-12

**Authors:** Katsuma Hayashi, Hiroshi Nishiura

**Affiliations:** Graduate School of Medicine, Kyoto University, Yoshidakonoecho, Sakyo-ku, Kyoto, 606-8501 Japan

**Keywords:** SARS-CoV2, Mortality, Lethality, Epidemiology, Age, Statistical estimation

## Abstract

**Background:**

It has been descriptively argued that the case fatality risk (CFR) of coronavirus disease (COVID-19) is elevated when medical services are overwhelmed. The relationship between CFR and pressure on health-care services should thus be epidemiologically explored to account for potential epidemiological biases. The purpose of the present study was to estimate the age-dependent CFR in Tokyo and Osaka over time, investigating the impact of caseload demand on the risk of death.

**Methods:**

We estimated the time-dependent CFR, accounting for time delay from diagnosis to death. To this end, we first determined the time distribution from diagnosis to death, allowing variations in the delay over time. We then assessed the age-dependent CFR in Tokyo and Osaka. In Osaka, the risk of intensive care unit (ICU) admission was also estimated.

**Results:**

The CFR was highest among individuals aged 80 years and older and during the first epidemic wave from February to June 2020, estimated as 25.4% (95% confidence interval [CI] 21.1 to 29.6) and 27.9% (95% CI 20.6 to 36.1) in Tokyo and Osaka, respectively. During the fourth wave of infection (caused by the Alpha variant) in Osaka the CFR among the 70s and ≥ 80s age groups was, respectively, 2.3 and 1.5 times greater than in Tokyo. Conversely, despite the surge in hospitalizations, the risk of ICU admission among those aged 80 and older in Osaka decreased. Such time-dependent variation in the CFR was not seen among younger patients < 70 years old. With the Omicron variant, the CFR among the 80s and older in Tokyo and Osaka was 3.2% (95% CI 3.0 to 3.5) and 2.9% (95% CI 2.7 to 3.1), respectively.

**Conclusion:**

We found that without substantial control, the CFR can increase when a surge in cases occurs with an identifiable elevation in risk—especially among older people. Because active treatment options including admission to ICU cannot be offered to the elderly with an overwhelmed medical service, the CFR value can potentially double compared with that in other areas of health care under less pressure.

**Supplementary Information:**

The online version contains supplementary material available at 10.1186/s12879-022-07929-8.

## Background

The coronavirus disease 2019 (COVID-19) pandemic has continued for more than 2 years, involving 620 million confirmed cases and over 6.5 million deaths worldwide as of 20 October 2022 [1]. The extended period of the pandemic was fuelled by the emergence of variants of concern, including the Alpha (B.1.1.7), Delta (B.1.617), and Omicron (B.1.1.529) variants, which were prevalent in Japan from March to May 2021, June to December 2021, and January 2022 onward, respectively; they had higher transmissibility and greater intrinsic severity than the wild type [2–7]. One of the most critical problems with COVID-19 was the surge in health-care demand, i.e. hospital caseload, which sometimes surpassed existing supply capacity in the midst of the pandemic: tragic deaths in the absence of access to health-care services were reported around the peak of the pandemic [8]. By the beginning of the Omicron wave in 2022, Japan had maintained a relatively low COVID-19 level compared with Western countries: less than 2% of cumulative risk of confirmed infection by the end of 2021; that helped health-care facilities offer essential care for all admitted patients [9, 10]. Tokyo and Osaka, two megacities in Japan, possess comparable and substantial capacity for testing and hospital admission, which enabled them to maintain health-care services for most of the pandemic period [9, 11]. However, because both are densely populated, they are always at risk of harbouring COVID-19 infection.

As a way of evaluating the risk of death, an epidemiological measurement of the severity of infection, i.e. the infection fatality risk (IFR)—defined as the risk of death among all infected individuals—is advantageous and free from ascertainment bias. However, the IFR calls for a sero-epidemiological survey or special estimation effort (e.g. repeated testing of randomly selected participants as practised in the United Kingdom); instead, the case fatality risk (CFR), using confirmed cases as the denominator, has been adopted in practice [12–16]. Studies indicate that the CFR of an entire population could be elevated when medical services are overwhelmed [17–23]; however, such analyses remain scarce.

When a medical service is overwhelmed, a prioritized service is instituted as part of triage—generally with younger patients with greater chance of survival being prioritized [24–26]. Nevertheless, little has been quantitatively studied during periods of the high demand for health-care services in Japan [11]. Even in Japan, where outbreak sizes have been smaller than in Western countries, the mortality clearly exceeded expected levels in 2021—especially when the epidemic was intense [27–30]. Specifically, severe COVID-19 cases in Osaka led to excessive intensive care unit (ICU) capacity from March to June 2021, with at least 19 patients dying at home [31]; however, that was not the case for Tokyo during the same period. In Japan, this particular period was the only point at which the health-care capacity for severe cases exceeded the available number of ICU beds in a number of areas [32].

**Fig. 1:**
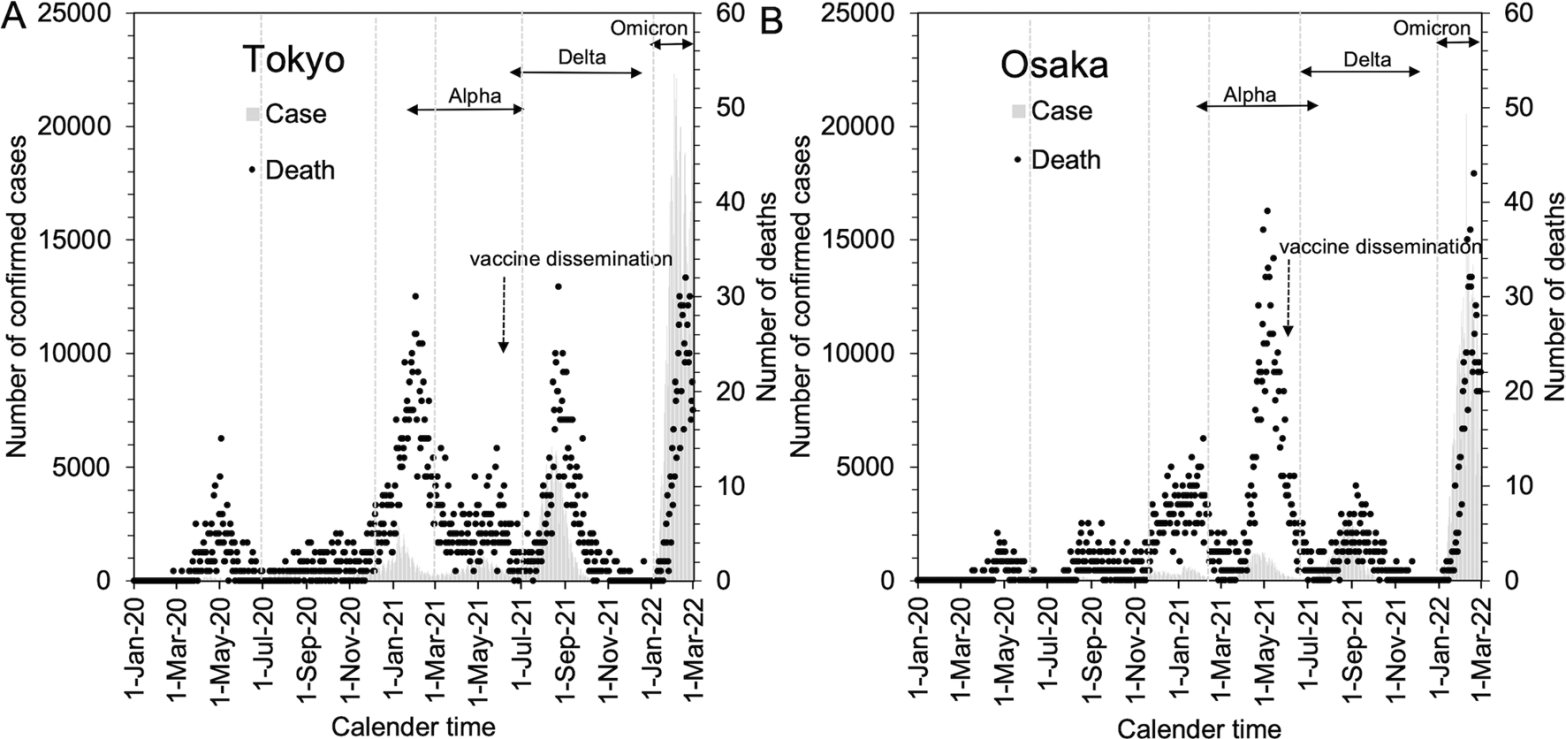
Epidemic curves of COVID-19 in Tokyo (**A**) and Osaka (**B**), 2020–2022. Grey bars represent reported number of confirmed cases, and dots indicate observed number of daily deaths as a function of the date of death. Vertical dashed lines separate the discrete time periods by pandemic wave, which we used to estimate the time-dependent case fatality risk as a step function. The lines indicate 1 June 2020, 1 December 2020, 1 March 2021, 1 July 2021, and 1 January 2022. Where there was a dominant variant of concern responsible for the epidemic wave, the arrows show the corresponding period. Vertical arrows indicate when vaccination started

The purpose of the present study was to estimate the age-dependent CFR in Tokyo and Osaka over time, investigating the impact of caseload demand on the risk of death. Comparing Tokyo and Osaka, which are geographically distant from each other (Fig. 1), we estimated the relative CFR, accounting for the time delay from diagnosis to death; moreover, the risk of ICU admission in Osaka was explored so that the impact of caseload demand on the CFR could be comprehensively clarified.

## Methods

### Epidemiological data

**Fig. 2:**
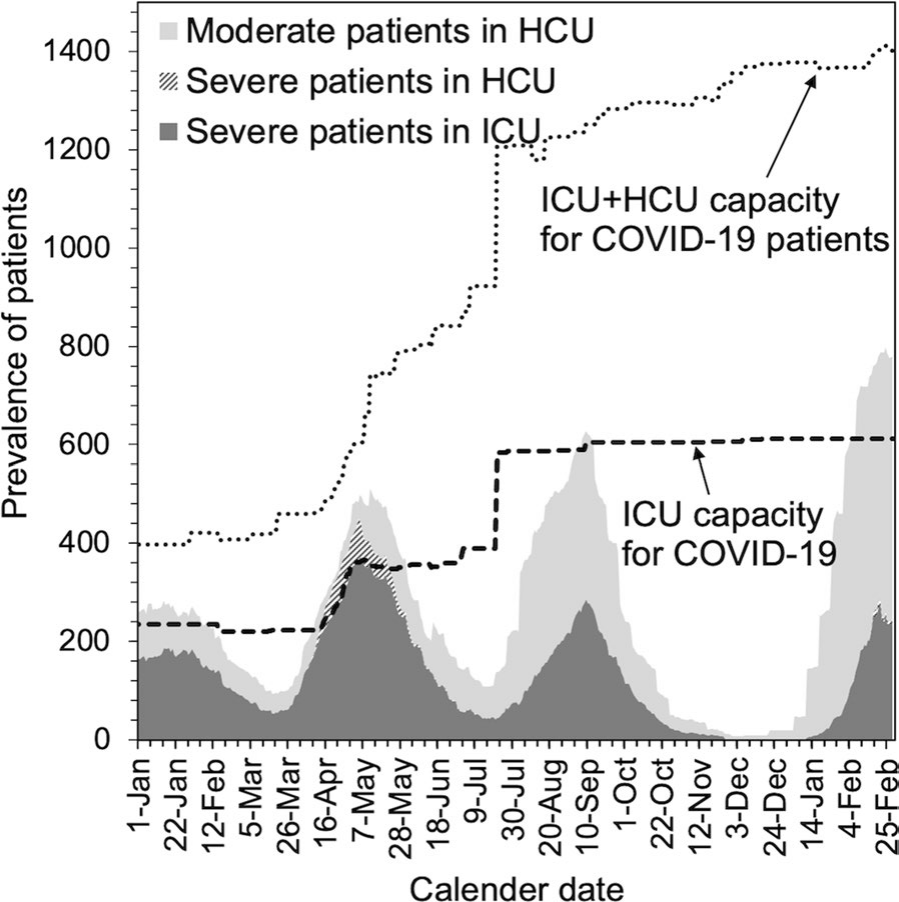
Changes in critical care beds in Osaka Prefecture. The dark-grey area shows the prevalence of severe cases admitted to intensive care units (ICUs) in Osaka Prefecture; the light-grey area indicates the prevalence of mild cases in high care units (HCUs) in Osaka Prefecture; and the shaded area shows the prevalence of severe patients in HUCs. Severe cases in Japan were defined as patients requiring ICU management or having respiratory failure and requiring mechanical ventilation. Mild cases were defined as patients who did not necessarily require ICU treatment. In Osaka Prefecture, the ICUs were saturated in the 2nd week of April 2021; the number of ICU patients started to decrease in the 1st week of May 2021. The thick dotted line indicates the number of available ICU beds at the time. The thin dotted line shows the number of available beds in ICUs and HCUs combined

In Japan, all laboratory-confirmed COVID-19 cases are mandatorily notified to the government following the Infectious Disease Control Law. Laboratory confirmation is made by real-time polymerase chain reaction. We retrieved the publicly announced number of confirmed cases and confirmed deaths from the Japanese government and the Tokyo and Osaka metropolitan governments [33, 34]. Specifically, the date of diagnosis, date of death, and age were obtained for the period 16 January 2020 to 1 March 2022. In Osaka, the incidence of severe cases (i.e. number of new severe cases) was also regularly announced: severe disease was defined as COVID-19 cases admitted to ICU or intubated to treat respiratory failure (Fig. 2). In this article, the risk of ICU admission is used to represent the summed risk, i.e. severe cases admitted to ICU and intubated.

### Statistical modelling

#### Estimating time from diagnosis to death

To estimate the CFR, while taking into account the right censoring, i.e., time delay from diagnosis to death, we first determined the time distribution from diagnosis to death, allowing for variations in the delays over time. To this end, a step function was employed to describe the mean and standard deviation (SD) of delay for each discrete time period (Table 1). We determined the time interval according to the pandemic wave: Japan had experienced six waves by 1 March 2022. Because confirmed patients aged 50 years or older are at risk of death, we ignored individuals younger than 50 years, and we made the evaluation for each 10-year age band *a*: 50–59 years, 60–69 years, 70–79 years, and 80 years and older. Among patients aged 40–49 years, only 42 deaths were observed from January to December 2021; that was partly due to the limited number of infections, and we were unable to obtain a stable estimate of the CFR as a function of time. Technical details of the estimation method for the delay distribution appear in the Additional file 2: Appendix.

**Table 1:** Discrete time period to estimate the time-dependent case fatality risk in Tokyo and Osaka, 2020–2022

$k$	Estimation periods	Dominant variant (if any)	Two dose vaccination coverage in Osaka (all ages/over 65 y.o.)	Two dose vaccination coverage in Tokyo (all ages/over 65 y.o.)
1	1st -Feb-2020–31-Jun-2020			
2	1st-Jul-2020–30-Nov-2020			
3	1st-Dec-2020–31-Feb-2021	Alpha		
4	1st-Mar-2021–31-Jun-2021	Alpha	10%/34%	10%/36%
5	1st-Jul-2021–31-Dec-2021	Delta	72%/91%	75%/91%
6	1st-Jan-2022–28-Feb-2022	Omicron(BA.1)	73%/91%	75%/91%

The vaccination coverages shown represent the reported number on the last date of the defined time intervals. The source of vaccination coverage is [35]

#### CFR estimation

We determined the CFR for each of the defined time intervals (by pandemic wave) described above. We estimated the parameters simultaneously for Tokyo and Osaka. In that way, we jointly determined the relative CFR in Osaka compared with Tokyo over the same time interval using the difference in delay from diagnosis to death between the two cities. Further details appear in the  Additional file 2: Appendix.

#### Risk of ICU admission by month in Osaka Prefecture

ICU admission risk is theoretically considered to reflect the clinical severity of disease. However, owing to limited medical capacity, patients requiring ICU treatment were not necessarily admitted there; thus, ICU admission risk could paradoxically decrease in such cases. By presenting the ICU admission risk chronologically together with the CFR, we examined time-series changes in hospital caseload pressure by comparing time-dependent changes in ICU admission risk and the CFR.

### Ethical considerations

This study was approved by the Medical Ethics Board of the Graduate School of Medicine at Kyoto University (R2676). The study used publicly available data only, having previously been de-identified.

### Data-sharing statement

The weekly number of confirmed cases and deaths in Tokyo and Osaka and the weekly number of confirmed severe cases in Osaka are available as Additional file 1. Data on deaths and infections are given for the first week as 5–11 January 2020. For severe cases, the 1st week is set as 6–12 December 2020. The R code used in this study is available upon request.

## Results

Table 2 shows the estimated time delay from diagnosis to death in Tokyo. This took 3–4 weeks from February 2020 to December 2021 among individuals aged 50–79 years. With increasing age, the time delay and variance shortened. When the Omicron wave started in January 2022, the time from diagnosis to death abruptly decreased across all age groups. Additional file 2: Fig. S1 visually confirms the log-normal distribution fit. In Osaka, the delay did not significantly deviate from that in Tokyo among people younger than 70 years. However, among those aged 80 years and older, there was a shorter delay than in Tokyo. The mean was 5.6 days (95% confidence interval [CI] − 8.0 to − 3.3), 6.0 days (95% CI − 7.6 to − 4.6), and 2.7 days (95% CI − 8.5 to − 0.4) shorter from December 2020 to February 2021, March to June 2021, and January to February 2022, respectively, compared with Tokyo.

**Table 2:** Number of days from diagnosis of COVID-19 to death in Tokyo, 2020–2022

	Age (years)							
Estimation period*	50–59		60–69		70–79		≥ 80	
	Mean	SD	Mean	SD	Mean	SD	Mean	SD
1	24.7	51.8	17.3	31.8	17.7	20.3	16.6	18.1
2	36.4	19.0	29.4	39.1	23.3	24.9	17.7	13.9
3	24.6	36.7	23.2	34.4	19.1	21.1	16.0	15.8
4	21.1	30.1	23.7	32.2	20.5	20.1	16.1	13.8
5	23.5	25.8	20.5	25.1	17.6	17.3	14.7	14.8
6	9.4	16.3	10.9	13.6	10.1	11.5	10.3	12.2

*The period of estimation corresponds to the epidemic waves in Japan. They were as follows: (1) 1 February 2020 to 30 June 2020; (2) 1 July 2020 to 30 November 2020; (3) 1 December 2020 to 28 February 2021; (4) 1 March 2022 to 30 June 2021; (5) 1 July 2021 to 31 December 2021; and (6) 1 January 2021 to 28 February 2022

Additional file 2: Table S1 summarizes the estimated CFR. As reported elsewhere, the CFR was always highest among the oldest age group. Moreover, the CFR was highest in most age groups during the first epidemic wave from February to June 2020. During the fifth wave (Delta variant) from July to December 2021, vaccination was under way; despite the increased severity of the Delta variant, the CFR tended to be lower than during earlier waves. The CFR during the sixth wave (Omicron variant) dropped sharply in all age groups. The relative CFR was not significantly different between Tokyo and Osaka among individuals younger than 70 years.

Among cases from the 70- to 79-year age group, the CFR in Osaka was 1.5 times (95% CI 1.1 to 1.9), 2.3 times (95% CI 1.9 to 2.7), and 2.1 times (95% CI 1.7 to 2.6) greater than in Tokyo from July to November 2020 (second wave), March to June 2021 (fourth wave), and January to February 2022 (sixth wave), respectively. Conversely, the CFR in Osaka in this age group was 0.6 times (95% CI 0.5 to 0.8) that in Tokyo from July to December 2021 (fifth wave).

**Fig. 3:**
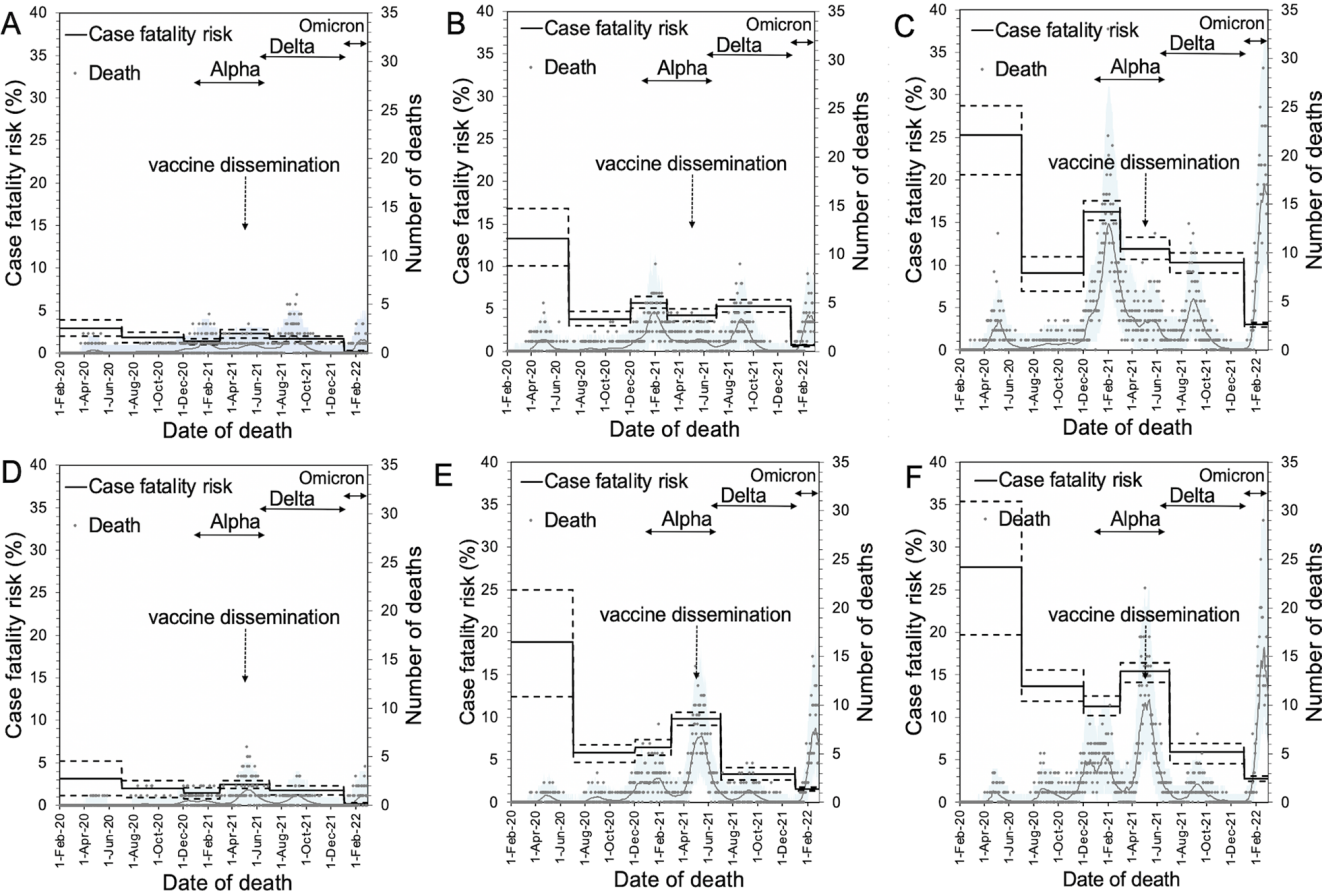
Case fatality risk and predicted number of deaths in Tokyo and Osaka, 2020–2022. **A**–**C** Show the case fatality risk (CFR) in Tokyo among the 60s, 70s, and 80 years and older age groups, respectively. **D**–**F** Indicate the CFR in Osaka among the 60s, 70s, and 80 years and older age groups, respectively. The dots show the observed number of daily deaths; the thin black curve indicates the expected number of deaths from our model. The light-grey shaded area represents the 95% confidence interval (CI) of daily deaths as computed by the parametric bootstrap method. The continuous step function shows the estimated CFR along with its 95% CI indicated as broken lines. If any dominant variant of concern was responsible, the horizontal arrow indicates the corresponding period. The CFR of the fourth wave (caused by the Alpha variant) yielded a higher CFR in Osaka than during the fifth wave. After the fifth wave (Delta variant), a substantial proportion of the population was protected

Among individuals 80 years and older, the CFR in Osaka was 1.5 times (95% CI 1.2 to 1.8) and 1.3 times (95% CI 1.1 to 1.5) higher than in Tokyo from July to November 2020 (second wave) and March to June 2021 (fourth wave), respectively. Conversely, the CFR in Osaka was 0.7 times (95% CI 0.6 to 0.8) and 0.6 times (95% CI 0.5 to 0.7) that in Tokyo from December 2020 to February 2021 (third wave) and July to December 2021 (fifth wave), respectively. Figure 3 illustrates the time-dependent trend of the CFR by age group in Tokyo and Osaka. The 95% CI of the predicted number of deaths contained the majority of observed data points, confirming that our model sufficiently captured the observed pattern.

**Fig. 4:**
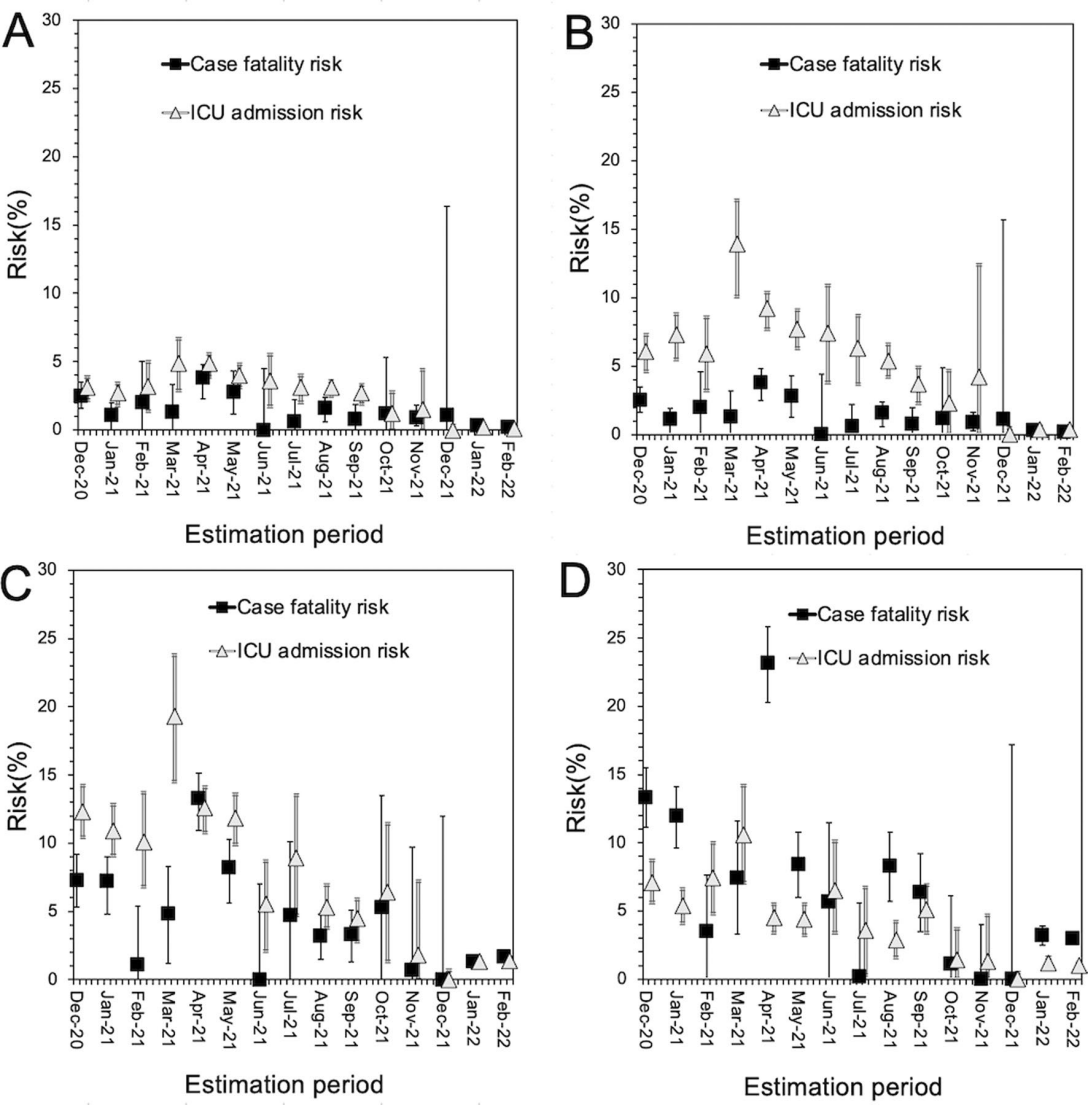
Relative risks of death and ICU admission as a function of time in Osaka, 2020–2022. **A**–**D** Show estimates among cases in the 50s, 60s, 70s, and 80 years and older age groups, respectively. The black filled squares show the relative CFR using the reference value of January 2020 as 1. Similarly, unfilled triangles indicate the relative risk of ICU admission. In **A** and **B**, the ICU admission risk was elevated in April and May 2021, but that in **C** and **D** decreased in the same period. The CFR in **C** and **D** was elevated during the corresponding time. The risk of ICU admission and the CFR behaved inversely among the elderly, indicating that the elevated CFR was not entirely due to decreased case ascertainment in the epidemic surge

Figure 4 compares the CFR and risk of ICU admission in Osaka as a function of time. It can be seen that the CFR is greater than ICU admission risk among individuals 80 years and older. As indicated by Fig. 2, the caseload demand exceeded the capacity in April and May 2021 in Osaka; physicians were forced to initiate triage to select younger severe cases for ICU admittance. Thus, the CFR among the 70 and 80s age groups was elevated. Conversely, the risk of ICU admission among those 80 years and older decreased when the caseload was overwhelming, reflecting the reduced active management service for the oldest patients. Among the 50 and 60s age groups, the risk of ICU admission increased during the same period.

## Discussion

The present study estimated the CFR in Tokyo and Osaka in real time to illuminate the impact of caseload demand for an overwhelmed medical service on the outcome of COVID-19. The CFR was highest among individuals aged 80 years and older; during the first wave, from February to June 2020, it was 25.4% and 27.9% in Tokyo and Osaka, respectively. With the Omicron variant, the CFR among those 80 years and older in Tokyo and Osaka was 3.2% (95% CI 3.0 to 3.5) and 2.9% (95% CI 2.7 to 3.1), respectively. During the fourth wave (caused by the Alpha variant), the interventions in Osaka were considerably delayed; the CFR among individuals in their 70 and 80s was 2.3 times and 1.5 times greater, respectively, than in Tokyo. Conversely, though a surge in hospitalizations occurred, the risk of ICU admission among the 80s and older age group decreased. Such time-dependent variation in the CFR was not seen among the younger group aged under 70 years.

An important result of this study is that the CFR can vary over time and may become elevated when ICU beds are scarce. This finding indicates that the overall outcomes for older individuals with an overwhelmed health-care service would be particularly worsened. In contrast to similar studies showing that the CFR rises when the number of infected patients increases, we have shown that the rise in CFR was not due to mere diagnostic bias: some age groups had lower ICU admission risk during the period when the CFR was on the rise [20, 36–38]. Japan prepared a relatively small number of hospital beds for COVID-19 patients compared with Western countries; this problem became evident when an apparent surge in cases occurred [11]. If caseload demand surpasses health-care capacity, its impact is evident first among elderly people. Thus, the limited resources of intensive care are prioritized for other rescuable patients, a proposition in line with previous findings [17–23]. Other possible reason for elevated CFR among the elderly is that the risk of clustering in care facilities (e.g. care homes) may be elevated; in that way, residents may be exposed to greater risk of death than community-dwelling older people [39–42]. Common to both explanations is that the elevated risk—upon increased incidence and under limited interventions—would certainly be more apparent in the elderly population [43–45].

A small number of studies have investigated the relationship between CFR and pressure on health-care services: they identified potential epidemiological biases, including ones associated with appropriate censoring, ascertainment, and reporting [46–48]. During a pandemic, following up a cohort of cases is very demanding, and it is common practice to estimate the CFR from cumulative counts of cases and deaths [15]. A few similar methods to our own for estimating the CFR in real time have been proposed and applied to other coronavirus diseases (severe acute respiratory syndrome and Middle East respiratory syndrome) and influenza [49, 50]. However, an additional complication has been the time dependence in the delay distribution from illness onset to death; that delay reportedly varies over time and space [51].

The CFR relationship between Tokyo and Osaka differed according to the pandemic wave. Osaka had a very high CFR during the fourth wave (caused by Alpha); the CFR in Tokyo was greater than in Osaka during the third wave (caused by the wild type) and the fifth wave (Delta). In these three waves, the surge in cases was initially seen in both prefectures. During the sixth wave (Omicron) from January to February 2022, the CFR in Osaka was 2.1 times that in Tokyo. There was available ICU capacity in this Omicron wave, but clustering of elderly cases was frequently observed in care facilities, and a substantial fraction of older adults remained unvaccinated [52].

Regarding the delay from diagnosis to death, we found that it could vary with time and space. Our result implies that the delay may be shortened when the CFR is elevated. For example, Additional file 2: Table S1 shows that the delay from diagnosis to death in the fourth and sixth waves among individuals aged 80 years or older in Osaka prefecture was clearly shorter than in Tokyo. This may reflect local epidemiological dynamics caused by an increase in the number of people who died at home or in elderly care facilities (where active supportive care was not necessarily available) as a result of the upsurge in hospitalized cases.

Another important point is that the causal relationship between the CFR and overwhelming health-care demand may require a well-designed causal model [53–55]. Although descriptive, the present study successfully showed that the CFR among the elderly was elevated and that their risk of ICU admission was lower during a period of pandemic surge. We regard our study as the first step towards characterizing the possible mechanism underlying elevation of the CFR, which we assessed by dynamic (time-dependent) estimation of the CFR over time, age, and space.

Several limitations deserve consideration. First, as described in many meta-analyses and other factor analysis studies on the CFR, we estimated it using a confirmed case count [38, 54, 56–68]. Confirmatory diagnosis involves ascertainment bias; the elevated CFR during the pandemic’s peak may partly be related to the lower frequency of diagnosis, whereas IFR and CFR diverged significantly [55, 69–71]. Second, the confirmed deaths could have been smaller than the actual total number of deaths. More bias-free measures, including excess mortality, should be adopted to determine precisely the disease burden of COVID-19 [27–30]. Third, we did not elucidate the mechanism for the development of specific and non-specific treatment. At the very least, the decreased CFR from the second wave implies that the gradually formulated treatment protocol may have contributed to lowering the CFR compared with the first wave. Fourth, identifying the mean delay from diagnosis to death required modelling, and verification of the validity still demands an analysis of registered case data (i.e. cohort observation of the course of infection). Fifth, it is necessary to evaluate pressure on a health-care service other than the risk of ICU admission: a causal investigation following the present study is required.

## Conclusion

This study has shown that the CFR can be elevated when a surge in cases occurs without substantial control; increased risk is more apparent among the elderly population. Active treatment options including ICU admission cannot be offered to the elderly with an overwhelmed medical service; thus, the CFR value could potentially double compared with that in other areas of health care under less pressure.

## Supplementary Information

**Additional file 1.** Data generated or analysed during this study.

**Additional file 2.** Appendix.

## Data Availability

The study used publicly available data only, having previously been de-identified (https://www.pref.osaka.lg.jp/iryo/osakakansensho/happyo.html). All data generated or analysed during this study are included in this published article and its Additional file 1.

## Abbreviations

COVID-19: Coronavirus disease 2019
CI: Confidence interval
CFR: Case fatality risk
ICU: Intensive care unit
IFR: Infection fatality risk
SD: Standard deviation
