# Supplementary material for: Time-dependent risk of COVID-19 death with overwhelmed health-care capacity in Japan, 2020–2022
Source: BMC Infect Dis. 2022 Dec 12;22:933. doi: 10.1186/s12879-022-07929-8 (PMC9744068; doi:10.1186/s12879-022-07929-8)
Supplement: Supplementary file 2 — Additional file 2. Appendix. [file 12879_2022_7929_MOESM2_ESM.docx]

Additional file

Time-dependent risk of COVID-19 death with overwhelmed health-care capacity in Japan, 2020–2021

By

Katsuma Hayashi, Hiroshi Nishiura

Contents:

1. Appendix
2. Figure S1
3. Figure S2
4. Figure S3
5. Table S1
6. Table S2
7. Table S3

## Appendix

### Estimating time from diagnosis to death

Let $f_{a,k,T,x}$ be the probability mass function of the delay from diagnosis to death of length *x* for age group *a* at time period *k* in Tokyo (*T*); the corresponding geometric mean $\mu_{a,k,T}$ and variance $\sigma_{a,k,T}$ were estimated using the maximum-likelihood method. We employed log-normal distribution because it fitted best among two-parameter distributions, i.e. gamma, Weibull, and log-normal:

| $f_{a,k,T,x}=\frac{1}{\sqrt{2\mu_{a,k,T}{\sigma_{a,k,T}}^{2}}x}\exp\left( \frac{{\left( \log(x \right)-\mu_{a,k,T})}^{2}}{2{\sigma_{a,k,T}}^{2}} \right)$ | (1) |
| --- | --- |

To avoid delayed announcement after death, we excluded the latest 3-week data from our analysis. The corresponding data were unavailable in Osaka; thus, we allowed the distribution in Osaka to have a mean of $\mu_{a,k,T}+e_{a,k}$ and share the same variance with Tokyo. The parameter $e_{a,k}$ was jointly estimated with the CFR.

### CFR estimation

Let the age-dependent CFR in Tokyo and Osaka be $q_{a,k,T}$ and $q_{a,k,O}$, respectively, which are jointly estimated. The relative CFR in Osaka in age group *a* and period *k* is $\varepsilon_{a,k}$, calculated as ${q_{a,k,O}/q}_{a,k,T}$. Supposing that the confirmed cases reported on day *t* in Tokyo and Osaka are $c_{a,t,T}$ and $c_{a,t,O}$, respectively, we have

| $E(d_{a,t,T})=\sum_{j=0}^{t} {q_{a,k(t-j),T}c}_{a,t-j,T}f_{a,k,T,j}$ | (2) |
| --- | --- |

where *k*(*t*−*j*) indicates the discrete time step to which the time *t*−*j* corresponds. It should be noted that the time-dependent CFR is inside the summation, so the value can vary as a function of the date on which cases were diagnosed. We assume that the observed number of deaths in Tokyo and Osaka was sufficiently characterized by Poisson distribution, i.e.

| $d_{a,t,T}\sim Poisson\left( \sum_{j=0}^{t} {q_{a,k(t-j),T}c}_{a,t-j,T}f_{a,k,T,j} \right)$ | (3) |
| --- | --- |

and

| $d_{a,t,O}\sim Poisson\left( \sum_{j=0}^{t} {q_{a,k(t-j),O}c}_{a,t-j,O}f_{a,k,O,j} \right).$ | (4) |
| --- | --- |

Let $\pi_{a,t,T}$ be an estimator of the CFR described by the right-hand side of equation (2), including unknown parameters $q_{a,k(t),T}$ that governed the outbreaks. Given the empirical data ***D***, the total likelihood to estimate the CFR was

| $L\left( q_{a,k,T},\varepsilon_{a,k},e_{a,k};\boldsymbol{D} \right)=\prod_{t,k} \frac{{\pi_{a,t,T}}^{m_{a,t,T}}e^{\left( -\pi_{a,t,T} \right)}}{m_{a,t,T}!}\prod_{t,k} \frac{\left( \pi_{a,t,O} \right)^{m_{a,t,O}}e^{\left( -\pi_{a,t,O} \right)}}{m_{a,t,O}!}$ | (5) |
| --- | --- |

where *m* signifies observed death count.

### Risk of ICU admission by month in Osaka Prefecture

We examined the incidence of ICU admission in Osaka from December 2020 to March 2022. We used the starting time of December 2020 because a substantial surge of severe cases began approximately in that month. Let $p_{a,h}$ be the risk of ICU admission in month *h*. Supposing that the incidence of severe cases in age group *a* at time *t* is $s_{a,t}$, we assumed that the incidence is sufficiently captured by Poisson distribution:

| $s_{a,t}\sim Poisson\left( \sum_{j=0}^{t} {p_{a,h}c}_{a,t-j}g_{a,j} \right)$ | (5) |
| --- | --- |

where *g* is the probability mass function of time delay from diagnosis to severe manifestation. We estimated ICU admission risk, assuming that the delay from admission to severe manifestation had a mean of 5 days and an SD of 5 days for the first to third waves, a mean of 4 days and SD of 4 days for the third to fifth waves for the Alpha or Delta variants, and a mean of 3 days and SD of 3 days for the sixth wave for the Omicron variant [41]. To evaluate the pressure on health-care facilities caused by caseload demand, we compared the ICU admission risk and the CFR estimated for every month *h*. For the calculation, we used the time-dependent $f_{a,k,T,x}$ as the time distribution from diagnosis to death in month *h*.

**Figure S1** Log-normal distribution for the delay distribution from diagnosis to death in Tokyo


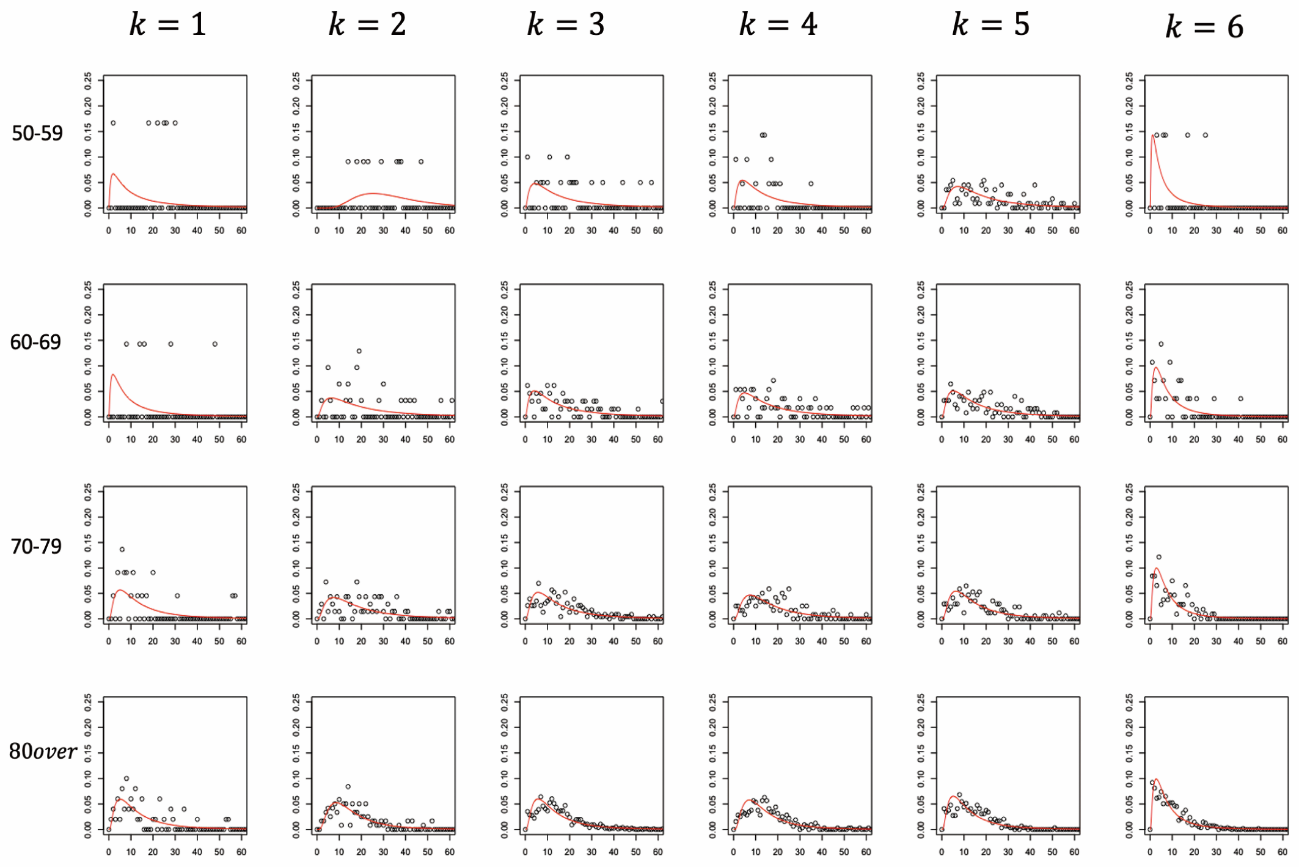


The top row is 50s, the second row 60s, the third row 70s, and the bottom row 80 years and older. *k* shows the estimation periods corresponding to Table 1. The vertical axis indicates the frequency, and the horizontal axis shows the number of days from diagnosis to death. The white circles indicate observed data, and the red line show fitting with a log-normal distribution. The parameters were estimated by maximum-likelihood estimation, and the estimates appear in Table 1.

**Figure S2** Case fatality risk and predicted number of deaths in Tokyo and Osaka, 2020–2022 few-parameters model


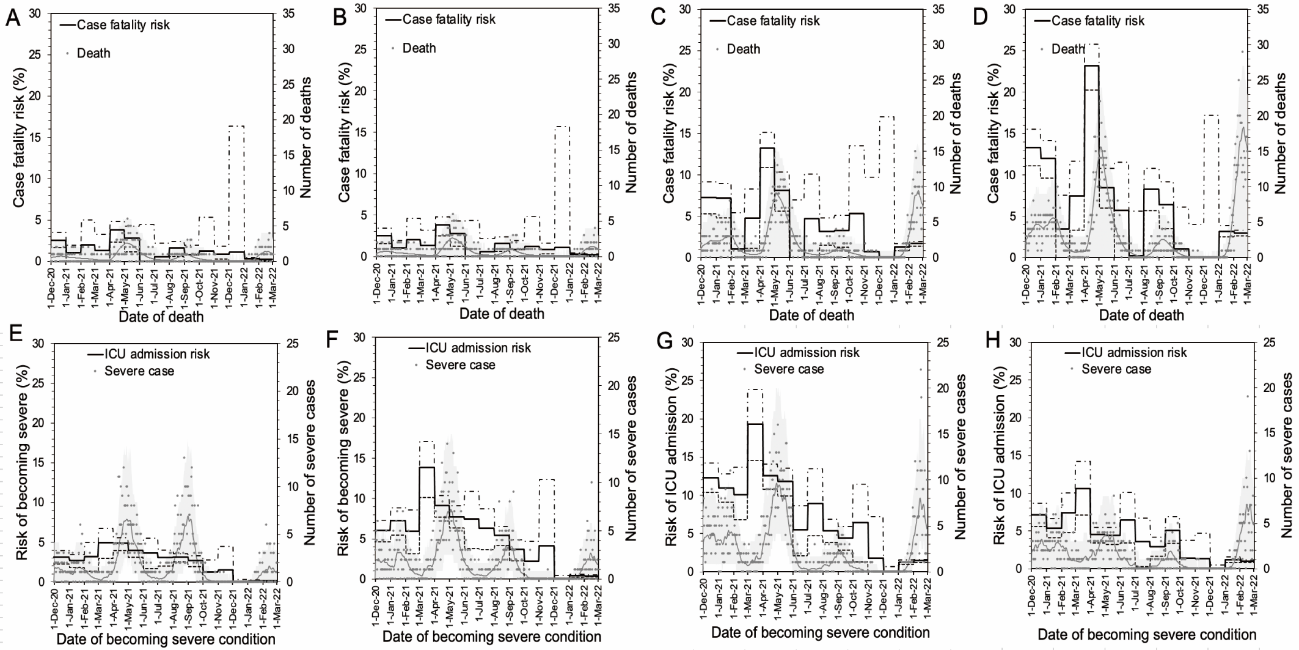


Panels A, B, and C show the case fatality risk (CFR) in Tokyo among the 60s, 70s, and 80 years and older age groups, respectively. Panels D, E, and F indicate the CFR in Osaka among the 60s, 70s, and 80 years and older age groups, respectively. The dots show the observed number of daily deaths; the thin black curve indicates the expected number of deaths from our model. The light-grey shaded area represents the 95% confidence interval (CI) of daily deaths as computed by the parametric bootstrap method. The continuous step function shows the estimated CFR along with its 95% CI indicates as broken lines. If a dominant variant of concern was responsible, the horizontal arrow indicates the corresponding period. The CFR of the fourth wave (caused by the Alpha variant) yielded a higher CFR in Osaka than during the fifth wave. From the fifth wave (Delta variant), a substantial proportion of the population was protected. Unlike in Fig. 3, the distribution from diagnosis to death was not estimated for Osaka Prefecture, but the parameters estimated for Tokyo were used as they appear here. Although the estimated CFR is slightly different from that in Fig. 3, the 95% CI sufficiently encompasses the observed data, and the data-fitted model does not visually deviate substantially from the data.

**Figure S3** Monthly estimation of risks of death and ICU admission and the predicted number of deaths in Osaka, 2020–2022


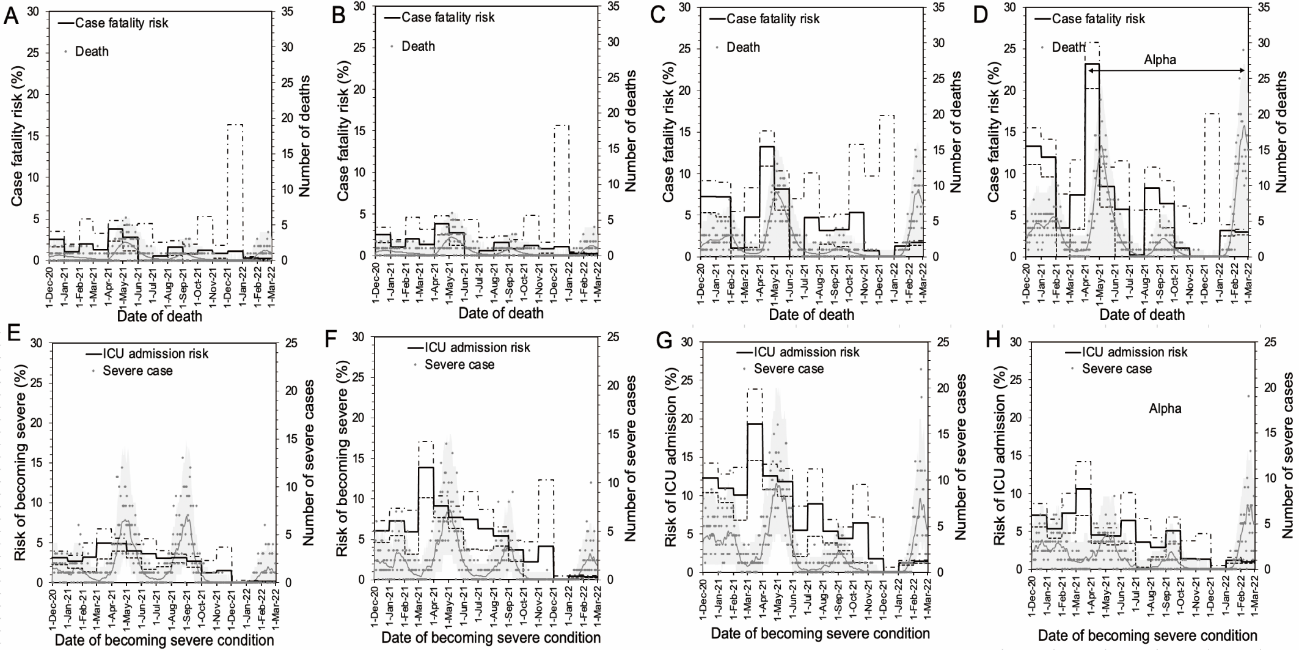


Panels A, B, C, and D show the case fatality risk (CFR) in Osaka among the 50s, 60s, 70s, and 80 years and older age groups, respectively. Panels E, F, G, and H indicate the risk of admission in Osaka among the 50s, 60s, 70s, and 80 years and older age groups, respectively. The dots show the observed number of daily deaths or severe cases; the thin black curve indicates the expected number of deaths or severe cases from our model. The light-grey shaded area represents the 95% confidence interval (CI) of daily deaths or severe cases as computed by the parametric bootstrap method. The continuous step function shows the estimated CFR or ICU admission risk along with its 95% CI indicated as broken lines.

Table S1. Estimated case fatality risk in Tokyo and Osaka, 2020–2022

|  | Tokyo | | Osaka | |  |  |  |  |
| --- | --- | --- | --- | --- | --- | --- | --- | --- |
|  | $q_{a,k,T}$:  CFR(%)  (95% CI) | | $q_{a,k,O}:$  CFR(%)  (95% CI) | | $\varepsilon_{a,k}$ (95% CI) | | $e_{a,k}$:Difference of means (days)  (95% CI) | |
| 50-59 years |  |  |  |  |  |  |  |  |
| 1st-Feb-2020 - 31-Jun-2020 | 0.5 | (0.5,0.6) | 0.5 | (0.0,1.1) | 1.0 | (0.0,2.2) | 1.0 | (-19.7,17.3) |
| 1st-Jul-2020 - 30-Nov-2020 | 0.5 | (0.2,0.8) | 0.5 | (0.0,0.6) | 1.0 | (0.0,2.5) | -5.0 | (-12.6,16.1) |
| 1st-Dec-2020 - 31-Feb-2021 | 0.5 | (0.0,1.3) | 0.5 | (0.0,0.6) | 1.0 | (0.0,4.9) | -1.0 | (-15.6,15.5) |
| 1st -Mar-2021 - 31-Jun-2021 | 0.6 | (0.5,0.7) | 0.6 | (0.5,0.7) | 1.0 | (0.7,1.3) | -5.0 | (-7.3,5.7) |
| 1st -Jul-2021 - 31-Dec-2021 | 0.7 | (0.5,0.9) | 0.7 | (0.4,0.9) | 1.0 | (0.4,1.7) | 9.0 | (-5.8,6.1) |
| 1st -Jan-2022 - 28-Feb-2022 | 0.1 | (0.0,0.3) | 0.1 | (0.0,0.1) | 1.0 | (0.0,3.5) | -4.0 | (-32.2,28.3) |
| 60-69 years |  |  |  |  |  |  |  |  |
| 1st-Feb-2020 - 31-Jun-2020 | 2.9 | (2.0,3.9) | 3.1 | (1.1,5.2) | 1.1 | (0.4,1.8) | 3.4 | (-12.8,12.7) |
| 1st-Jul-2020 - 30-Nov-2020 | 1.9 | (1.2,2.4) | 2.0 | (0.9,2.9) | 1.1 | (0.5,1.6) | -1.0 | (-13.3,12.5) |
| 1st-Dec-2020 - 31-Feb-2021 | 1.4 | (1.0,1.7) | 1.4 | (0.8,2.1) | 1.1 | (0.5,1.6) | -1.0 | (-16.4,17.2) |
| 1st -Mar-2021 - 31-Jun-2021 | 2.3 | (1.8,2.8) | 2.5 | (2.0,2.9) | 1.1 | (0.8,1.3) | -1.8 | (-5.8,5.5) |
| 1st -Jul-2021 - 31-Dec-2021 | 1.7 | (1.3,2.0) | 1.8 | (1.2,2.3) | 1.1 | (0.6,1.6) | -1.7 | (-8.5,8.1) |
| 1st -Jan-2022 - 28-Feb-2022 | 0.2 | (0.1,0.3) | 0.2 | (0.1,0.4) | 1.0 | (0.8,1.2) | 0.9 | (-4.5,5.0) |
| 70-79 years |  |  |  |  |  |  |  |  |
| 1st-Feb-2020 - 31-Jun-2020 | 13.3 | (10.1,16.8) | 18.9 | (12.4,25.0) | 1.4 | (0.9,2.1) | 2.0 | (-3.5,10.0) |
| 1st-Jul-2020 - 30-Nov-2020 | 3.8 | (3.0,4.7) | 5.8 | (4.7,6.8) | 1.5 | (1.1,1.9) | -2.6 | (-8.3,3.1) |
| 1st-Dec-2020 - 31-Feb-2021 | 5.8 | (5.1,6.5) | 6.5 | (5.6,7.4) | 1.1 | (0.9,1.4) | -2.3 | (-5.5,1.8) |
| 1st -Mar-2021 - 31-Jun-2021 | 4.3 | (3.6,5.0) | 9.8 | (9.1,10.6) | 2.3 | (1.9,2.7) | 0.0 | (-2.3,2.0) |
| 1st -Jul-2021 - 31-Dec-2021 | 5.3 | (4.6,6.1) | 3.3 | (2.6,4.1) | 0.6 | (0.5,0.8) | -0.5 | (-4.9,4.1) |
| 1st -Jan-2022 - 28-Feb-2022 | 0.7 | (0.6,0.9) | 1.6 | (1.3,1.8) | 2.1 | (1.7,2.6) | 0.2 | (-1.8,3.0) |
| 80 over years |  |  |  |  |  |  |  |  |
| 1st-Feb-2020 - 31-Jun-2020 | 25.4 | (21.1,29.6) | 27.9 | (20.6,36.1) | 1.1 | (0.8,1.5) | -2.2 | (-5.8,1.0) |
| 1st-Jul-2020 - 30-Nov-2020 | 9.4 | (7.9,10.9) | 13.9 | (12.0,16.2) | 1.5 | (1.2,1.8) | -3.0 | (-6.2,0.3) |
| 1st-Dec-2020 - 31-Feb-2021 | 16.3 | (15.3,17.3) | 11.2 | (10.0,12.4) | 0.7 | (0.6,0.8) | -5.6 | (-8.0,-3.3) |
| 1st -Mar-2021 - 31-Jun-2021 | 11.5 | (10.5,13.1) | 15.3 | (14.1,16.5) | 1.3 | (1.1,1.5) | -6.0 | (-7.6,-4.6) |
| 1st -Jul-2021 - 31-Dec-2021 | 10.3 | (9.2,11.4) | 5.9 | (4.7,6.9) | 0.6 | (0.5,0.7) | -2.9 | (-7.0,1.9) |
| 1st -Jan-2022 - 28-Feb-2022 | 3.2 | (3.0,3.5) | 2.9 | (2.7,3.1) | 0.9 | (0.8,1.0) | -2.7 | (-5.0,-0.4) |

CFR, case fatality risk; $\varepsilon_{a,k}$, the relative case fatality risk in Osaka compared to that in Tokyo; $e_{a,k}$, added mean time delay (days) from diagnosis to death in Osaka compared to that in Tokyo. Numbers in parentheses represent the 95% confidence intervals computed by means of the parametric bootstrap method.

Table S2. Comparison of estimates in the 18-parameter model with those in the 12-parameter model

|  | Tokyo | | | | Osaka | | | |
| --- | --- | --- | --- | --- | --- | --- | --- | --- |
|  | 18 parameters model | | 12 parameters model | | 18 parameters model | | 12 parameters model | |
|  | CFR(%) (95% CI) | | CFR(%) (95% CI) | | CFR(%) (95% CI) | | CFR(%) (95% CI) | |
| 50-59 |  | |  | |  | |  | |
| 1st-Feb-2020 - 31-Jun-2020 | 0.5 | (0.5,0.6) | 0.6 | (0.4,0.9) | 0.5 | (0.0,1.1) | 0.7 | (0.0,1.5) |
| 1st-Jul-2020 - 30-Nov-2020 | 0.5 | (0.2,0.8) | 0.5 | (0.3,0.6) | 0.5 | (0.0,0.6) | 0.5 | (0.1,0.7) |
| 1st-Dec-2020 - 31-Feb-2021 | 0.5 | (0.0,1.3) | 0.2 | (0.2,0.2) | 0.5 | (0.0,0.6) | 0.2 | (0.1,0.4) |
| 1st -Mar-2021 - 31-Jun-2021 | 0.6 | (0.5,0.7) | 0.7 | (0.5,0.9) | 0.6 | (0.5,0.7) | 0.7 | (0.5,0.9) |
| 1st -Jul-2021 - 31-Dec-2021 | 0.7 | (0.5,0.9) | 0.6 | (0.5,0.7) | 0.7 | (0.4,0.9) | 0.6 | (0.4,0.7) |
| 1st -Jan-2022 - 28-Feb-2022 | 0.1 | (0.0,0.3) | 0.1 | (0.1,0.1) | 0.1 | (0.0,0.1) | 0.1 | (0.1,0.1) |
| 60-69 |  |  |  |  |  |  |  |  |
| 1st-Feb-2020 - 31-Jun-2020 | 2.9 | (2.0,3.9) | 2.9 | (2,3.8.0) | 3.1 | (1.1,5.2) | 3.1 | (1.1,5.3) |
| 1st-Jul-2020 - 30-Nov-2020 | 1.9 | (1.2,2.4) | 1.9 | (1.2,2.5) | 2.0 | (0.9,2.9) | 2.0 | (1.2,2.8) |
| 1st-Dec-2020 - 31-Feb-2021 | 1.4 | (1.0,1.7) | 1.3 | (1.0,1.7) | 1.4 | (0.8,2.1) | 1.4 | (0.8,2.0) |
| 1st -Mar-2021 - 31-Jun-2021 | 2.3 | (1.8,2.8) | 2.3 | (1.9,2.8) | 2.5 | (2.0,2.9) | 2.5 | (2.0,2.9) |
| 1st -Jul-2021 - 31-Dec-2021 | 1.7 | (1.3,2.0) | 1.7 | (1.3,2.0) | 1.8 | (1.2,2.3) | 1.8 | (1.1,2.3) |
| 1st -Jan-2022 - 28-Feb-2022 | 0.2 | (0.1,0.3) | 0.2 | (0.1,0.3) | 0.2 | (0.1,0.4) | 0.2 | (0.1,0.3) |
| 70-79 |  |  |  |  |  |  |  |  |
| 1st-Feb-2020 - 31-Jun-2020 | 13.3 | (10.1,16.8) | 14.4 | (11,17.2) | 18.9 | (12.4,25.0) | 15.3 | (10.6,20.9) |
| 1st-Jul-2020 - 30-Nov-2020 | 3.8 | (3.0,4.7) | 4.5 | (3.6,5.5) | 5.8 | (4.7,6.8) | 6.1 | (4.8,7.2) |
| 1st-Dec-2020 - 31-Feb-2021 | 5.8 | (5.1,6.5) | 6.1 | (5.3,6.7) | 6.5 | (5.6,7.4) | 5.9 | (5.0,6.8) |
| 1st -Mar-2021 - 31-Jun-2021 | 4.3 | (3.6,5.0) | 3.8 | (3.0,4.6) | 9.8 | (9.1,10.6) | 10.0 | (8.9,10.8) |
| 1st -Jul-2021 - 31-Dec-2021 | 5.3 | (4.6,6.1) | 4.8 | (4.2,5.5) | 3.3 | (2.6,4.1) | 3.8 | (2.7,4.6) |
| 1st -Jan-2022 - 28-Feb-2022 | 0.7 | (0.6,0.9) | 0.6 | (0.5,0.8) | 1.6 | (1.3,1.8) | 1.6 | (1.5,1.8) |
| 80 over |  |  |  |  |  |  |  |  |
| 1st-Feb-2020 - 31-Jun-2020 | 25.4 | (21.1,29.6) | 25.5 | (21.2,29.1) | 27.9 | (20.6,36.1) | 28.0 | (18.8,37.6) |
| 1st-Jul-2020 - 30-Nov-2020 | 9.4 | (7.9,10.9) | 10.1 | (8.3,11.9) | 13.9 | (12.0,16.2) | 16.1 | (14.2,18.2) |
| 1st-Dec-2020 - 31-Feb-2021 | 16.3 | (15.3,17.3) | 16.6 | (15.4,17.6) | 11.2 | (10.0,12.4) | 10.4 | (9.3,11.5) |
| 1st -Mar-2021 - 31-Jun-2021 | 11.5 | (10.5,13.1) | 11.0 | (9.7,12.2) | 15.3 | (14.1,16.5) | 15.1 | (14.0,16.2) |
| 1st -Jul-2021 - 31-Dec-2021 | 10.3 | (9.2,11.4) | 10.1 | (9.1,11.1) | 5.9 | (4.7,6.9) | 5.5 | (4.5,6.7) |
| 1st -Jan-2022 - 28-Feb-2022 | 3.2 | (3.0,3.5) | 3.2 | (3,3.5) | 2.9 | (2.7,3.1) | 3.0 | (2.8,3.3) |

The 18-parameter model corresponds to Fig. 3. The 12-parameter model corresponds to Fig. S2 and there is no substantial difference between the two models. The AICs for the 12- and 18-parameter models are 6012.3 and 6149.7, 331.7 for the over 80s, 349.4 for the 70s, 1723.9 and 1712.3 for the 60s, and 1323.0 and 1314.0 for the 50s.

Table S3. Estimated case fatality risk and ICU admission risk in Osaka, 2020–2022

| Age(years) | CFR(%) (95% CI) | | Risk of ICU Admission(%) (95% CI) | | Age(years) | CFR(%) (95% CI) | | Risk of ICU Admission(%) (95% CI) | |
| --- | --- | --- | --- | --- | --- | --- | --- | --- | --- |
| 50-59 |  |  |  |  | 70-79 |  |  |  |  |
| Dec-2020 | 2.5 | (1.6,3.5) | 3.1 | (2.2,3.9) | 2020-Dec | 7.3 | (5.3,9.2) | 12.3 | (10.4,14.2) |
| Jan-2021 | 1.1 | (0.0,2) | 2.7 | (1.8,3.4) | 2021-Jan | 7.2 | (4.8,9) | 10.9 | (9.1,12.8) |
| Feb-2021 | 2.0 | (0.0,5) | 3.2 | (1.4,5) | 2021-Feb | 1.1 | (0.0,5.4) | 10.1 | (6.8,13.7) |
| Mar-2021 | 1.3 | (0.0,3.3) | 4.9 | (2.9,6.7) | 2021-Mar | 4.8 | (1.2,8.3) | 19.3 | (14.5,23.8) |
| Apr-2021 | 3.8 | (2.3,4.8) | 4.9 | (3.9,5.6) | 2021-Apr | 13.3 | (10.9,15.1) | 12.6 | (10.8,14.1) |
| May-2021 | 2.8 | (1.2,4.3) | 4.0 | (3.1,4.8) | 2021-May | 8.2 | (5.6,10.3) | 11.8 | (9.9,13.6) |
| Jun-2021 | 0.0 | (0.0,4.5) | 3.6 | (1.7,5.5) | 2021-Jun | 0.0 | (0.0,7) | 5.5 | (2.1,8.7) |
| Jul-2021 | 0.6 | (0.0,2.2) | 3.1 | (2,4) | 2021-Jul | 4.7 | (0.0,10.1) | 8.9 | (4.7,13.5) |
| Aug-2021 | 1.6 | (0.6,2.4) | 3.1 | (2.5,3.6) | 2021-Aug | 3.2 | (1.5,4.8) | 5.3 | (3.8,6.9) |
| Sep-2021 | 0.8 | (0.0,1.9) | 2.7 | (1.9,3.3) | 2021-Sep | 3.3 | (1.3,5.1) | 4.5 | (2.8,5.9) |
| Oct-2021 | 1.2 | (0.0,5.3) | 1.2 | (0.0,2.8) | 2021-Oct | 5.3 | (0.0,13.5) | 6.4 | (1.3,11.4) |
| Nov-2021 | 0.9 | (0.3,1.8) | 1.5 | (0.0,4.4) | 2021-Nov | 0.7 | (0.0,9.7) | 1.8 | (0.0,7.2) |
| Dec-2021 | 1.1 | (0.0,16.4) | 0.0 | (0.0,0.3) | 2021-Dec | 0.0 | (0.0,47.3) | 0.0 | (0.0,0.7) |
| Jan-2022 | 0.3 | (0.1,0.4) | 0.2 | (0.1,0.2) | 2022-Jan | 1.3 | (0.8,1.7) | 1.3 | (0.9,1.6) |
| Feb-2022 | 0.2 | (0.1,0.3) | 0.1 | (0.1,0.2) | 2022-Feb | 1.7 | (1.4,2) | 1.4 | (1.2,1.6) |
| 60-69 |  |  |  |  | 80 and more |  |  |  |  |
| Dec-2020 | 2.5 | (1.6,3.5) | 6.1 | (4.6,7.3) | 2020-Dec | 13.3 | (11.1,15.5) | 7.1 | (5.6,8.7) |
| Jan-2021 | 1.1 | (0.0,1.9) | 7.3 | (5.5,8.8) | 2021-Jan | 12.0 | (9.6,14.1) | 5.4 | (4.1,6.6) |
| Feb-2021 | 2.0 | (0.0,4.6) | 5.9 | (3.2,8.6) | 2021-Feb | 3.5 | (0.0,7.6) | 7.4 | (4.8,10) |
| Mar-2021 | 1.3 | (0.0,3.2) | 13.9 | (10.1,17.1) | 2021-Mar | 7.4 | (3.3,11.6) | 10.6 | (7.1,14.2) |
| Apr-2021 | 3.8 | (2.5,4.8) | 9.2 | (7.7,10.4) | 2021-Apr | 23.2 | (20.3,25.8) | 4.5 | (3.4,5.5) |
| May-2021 | 2.8 | (1.3,4.3) | 7.7 | (6.3,9.1) | 2021-May | 8.4 | (6,10.8) | 4.4 | (3.2,5.5) |
| Jun-2021 | 0.0 | (0.0,4.4) | 7.4 | (3.8,10.9) | 2021-Jun | 5.7 | (0.0,11.5) | 6.5 | (3.4,10.1) |
| Jul-2021 | 0.6 | (0.0,2.2) | 6.3 | (3.7,8.7) | 2021-Jul | 0.2 | (0.0,5.6) | 3.6 | (0.3,6.7) |
| Aug-2021 | 1.6 | (0.6,2.4) | 5.4 | (4.2,6.6) | 2021-Aug | 8.3 | (5.7,10.8) | 2.9 | (1.6,4.2) |
| Sep-2021 | 0.8 | (0.0,2) | 3.7 | (2.3,4.9) | 2021-Sep | 6.4 | (3.5,9.2) | 5.1 | (3.4,6.9) |
| Oct-2021 | 1.2 | (0.0,4.9) | 2.3 | (0.0,4.7) | 2021-Oct | 1.1 | (0.0,6.1) | 1.4 | (0.0,3.7) |
| Nov-2021 | 0.9 | (0.3,1.6) | 4.2 | (0.0,12.4) | 2021-Nov | 0.0 | (0.0,4) | 1.3 | (0.0,4.7) |
| Dec-2021 | 1.1 | (0.0,15.7) | 0.0 | (0.0,0.5) | 2021-Dec | 0.0 | (0.0,17.2) | 0.0 | (0.0,0.5) |
| Jan-2022 | 0.3 | (0.1,0.5) | 0.4 | (0.2,0.6) | 2022-Jan | 3.2 | (2.5,3.9) | 1.2 | (0.8,1.6) |
| Feb-2022 | 0.2 | (0.1,0.3) | 0.4 | (0.3,0.5) | 2022-Feb | 3.0 | (2.6,3.3) | 1.0 | (0.8,1.2) |

Parameter estimates corresponding to Fig. 4, estimated monthly from December 2020 to the end of February 2022. 95% confidence intervals were calculated by parametric bootstrapping.
